# Systematic Review on Which Analytics and Learning Methodologies Are Applied in Primary and Secondary Education in the Learning of Robotics Sensors

**DOI:** 10.3390/s21010153

**Published:** 2020-12-29

**Authors:** Daniel Amo, Paul Fox, David Fonseca, César Poyatos

**Affiliations:** 1 Group of Research GRETEL, Engineering Department, La Salle, Ramon Llull University, 08022 Barcelona, Spain; 2 Group of Research GRETEL, Management Department, La Salle, Ramon Llull University, 08022 Barcelona, Spain; paul.fox@salle.url.edu; 3 Group of Research GRETEL, Architecture Department, La Salle, Ramon Llull University, 08022 Barcelona, Spain; 4 Group of Research EDI, Didactics and Theory of Education Department, Autonomous University of Madrid, 28049 Madrid, Spain; cesar.poyatos@uam.es

**Keywords:** learning analytics, primary and secondary education, robotic sensors, educational robotics, systematic review, STE(A)M, PRISMA methodology

## Abstract

Robotics technology has become increasingly common both for businesses and for private citizens. Primary and secondary schools, as a mirror of societal evolution, have increasingly integrated science, technology, engineering and math concepts into their curricula. Our research questions are: “In teaching robotics to primary and secondary school students, which pedagogical-methodological interventions result in better understanding and knowledge in the use of sensors in educational robotics?”, and “In teaching robotics to primary and secondary school students, which analytical methods related to Learning Analytics processes are proposed to analyze and reflect on students’ behavior in their learning of concepts and skills of sensors in educational robotics?”. To answer these questions, we have carried out a systematic review of the literature in the Web of Science and Scopus databases regarding robotics sensors in primary and secondary education, and Learning Analytics processes. We applied PRISMA methodology and reviewed a total of 24 articles. The results show a consensus about the use of the Learning by Doing and Project-Based Learning methodologies, including their different variations, as the most common methodology for achieving optimal engagement, motivation and performance in students’ learning. Finally, future lines of research are identified from this study.

## 1. Introduction

The acronym, STEM refers to science, technology, engineering, and math. The National Science Foundation initially began to use the acronym SMET (for science, math, engineering, and technology) but decided to change it to STEM for phonetic reasons. The evolution of the term STEM in education has led some authors to create the concepts “STEM Education”, “Integrative STEM Education”, and “STEM Integration” [1]. STEM Education has been defined in different ways and from different disciplines [2,3,4,5], “is used to identify individual subjects, a stand-alone course, a sequence of courses, activities involving any of the four areas, a STEM-related course, or an interconnected or integrated program of study” [6]. Integrative STEM Education is a dynamic teaching-learning process focused on students. STEM Integration is a more static process overseen by the teacher [7], as an approach where the borders between the different disciplines are blurred through a progressive integration that implies a greater interconnection and interdependence between disciplines [4]. Anywise, authentic STEM education should increase students’ understanding of how things work and improve their use by different technologies.

STEM [8,9] skills can contribute to the empowerment of youth by eliminating the gender gap in education [10,11,12,13] and by providing equal employment opportunities as a strategy to reduce overall inequalities, eradicate poverty and promote peace and prosperity for all. Moreover, many nations propose improving STEM education as a response to increasing demand for STEM skills to meet economic challenges [3,14,15]. These are all challenges posed in the 2030 Agenda [16,17,18]. Therefore, STEM education or STEM integration in pre-university education can help future engineers to solve current and future sustainability problems [19,20]. However, integrating STEM in primary and secondary education needs a well-defined framework for instructional practices [21,22,23]. Our intention in this paper is to focus on any kind of STEM education, searching for those teaching-learning experiences, with a particular focus on robotics sensors, that could help understand which strategies are better to adopt.

All of the papers presented in our literature review seek to engage and prepare our students to be future leaders and effective members of our global society [16,24]. In fact, STEM is currently widely used in primary and secondary education curricula primarily through the use of robotics to teach key science-based concepts [25,26,27]. Hence, robotics is growing in importance in pre-university education. Furthermore, it has the power to motivate young students, either as a field of knowledge in itself to learn complex notions in an almost play-like environment, or as a tool to present technology and other subjects to those students in an attractive and motivating manner [28,29].

This reflects the fact that robotics technologies have become increasingly common in both business and private life. These include such examples as advanced Artificial Intelligence (AI) for facial recognition [30,31,32,33], industrial robotics, and automation in society as well as autonomous and self-driving cars, aerial drones, or the integration of robots in the workplace and in manufacturing [34,35,36]. Moreover, robots are expected to perform the work of about 800 million employees by 2030 [28,37,38]. Policymakers have understood that adding the teaching of scientific concepts to these examples is essential to addressing the future needs of society and industry [39]. Therefore, STEM education is critical to addressing the future needs of a technology-driven and sustainable global economy [16,40,41,42]. STEM education can help handle possible future adaptation issues by introducing Artificial Intelligence and robotics [43], two science fields that fits well in conjunction.

Robotics is an innovative field that embraces different scientific domains, from physics and electronics to mechanical engineering, mathematics, and computer programming [44,45,46,47]. Educational robotics (ER) is robotics applied to education to teach STEM through activities that use simple as well as complex robots [48,49,50].

The constructivist learning theory proposed by Piaget and Papert [35,51,52,53,54] is the basis of ER. In an ER activity or lesson, students design, build, program, debug and share their robotic constructions. When students create these personal and expressive robots, they construct their own unique meanings for concepts. Furthermore, ER can also support inclusive education in computer science and robotics literacy for all ages [55,56], as demonstrated by the Crumble robot workshops, for instance [57].

Constructivism [58,59], as an educational method based on the constructivist learning theory, is one of the reasons that ER is integrated into primary and secondary education. Constructing a robot is considered an integral part of the learning process, where the creativity and enthusiasm of students are stimulated through an open-ended and problem-solving process in the real world. Regarding STEM, working with ER in pre-university education instills technological literacy and a better understanding of the different parts that make up a robot as an engineering system [52].

Hence, constructivist learning theory is a cornerstone of ER teaching and learning processes. Moreover, understanding the way the brain works is necessary to inspire new scientists by helping them to think smart, be sensible, and wise [60]. Most students receive almost no education in neuroscience, and there is no public understanding of the brain. Educational neurorobotics [25,61], where neurorobotics is the study of robots controlled by artificial nervous systems, has the potential to revolutionize STEM education and the understanding of the brain [25,62].

In ER curricula, as extracted from our literature review, some of the most effective methodologies to learn STEM concepts and skills are learning by doing and project-based learning approaches [63], which facilitate the implementation of robotics technology in an interdisciplinary way. Project-based learning is student-centered, focused on a specific topic, driven by a set of accomplishments to achieve, and usually finishes with a robotic construction. Projects foster an environment of discussion, creativity, problem-solving, inquiry, modeling, and testing, and may be applied to all grade levels and subjects, such as programming. For instance, combining ER with visual programming makes it more attractive, fosters students’ attention and interest, and results in immediate feedback [64,65,66,67].

Robotics competitions are another scenario that has become widely available around the world in recent years [68,69,70,71,72]. This scenario can incorporate different types of robotics frameworks and stimulate interest in robotics in primary and secondary students. Schools learn by using the ER curriculum, subjects, material, and classrooms to practice for these events. Furthermore, practice for robotic competitions involves and promotes STEM. Students need to collaborate, communicate, and use skills beyond science-based ones to solve competition challenges [28].

To support constructivist learning, there are many robotics commercial based kits available to teach STEM. They are usually composed of particular robotic hardware which is programmed in a particular language using a particular software coding tool that only works in a particular commercial based kit suite. Each vendor usually has its own hardware and software that is incompatible with each other, with popular programming languages, such as python, and standard sensors. With such basic hardware, commercial based kits are difficult to modify in their mechanical and electronic structure [73] so new types of sensors can rarely be introduced to expand learning or AI concepts [43]. Therefore, most of them are limited to learning basic skills. Usually, there is only time in the classroom to finish hardware and mechanical assembly [74], and the robots that students can construct are limited to a few possible applications quite far from reality [75]. Besides, for many schools in developing countries, the acquisition of these toolkits could become an economical barrier [73]. Hence, some authors remark that it is important to integrate open robotics frameworks [73] and to incorporate other kinds of robotic sensors, such as vision systems [28].

In the ER context, the teaching of robotics converges with other disciplines and fields such as learning analytics [76]. In the case of learning analytics, the aim is to comprehend how students behave in the use of robotics to enhance learning processes. Siemens, which advocates connectivism, is the first to enter into the academic debate to define Learning Analytics from a pedagogical perspective [77]. To the definitions of that time [78], he adds data beyond the virtual learning environment (VLE), such as social networks or personal blogs. He describes Learning Analytics as “the use of intelligent data, learner-produced data, and analysis models to discover information and social connections, and to predict and advise on learning” [77]. Siemens’ definition is broader in the sense that it fits the educational system and complements it. His description, in his own words, “is less clean, but it does not try to modify the educational system”, but rather to use the analytical results to improve it. Therefore, Learning Analytics in ER helps to improve teaching and learning science-based and art concepts and skills [67,78,79,80,81,82]. However, we found only two references that converge ER, robotics sensors, and Learning Analytics [51,65]. Notwithstanding the few references, the positive results in both pieces of research foster to continue integrating Learning Analytics in the teaching and learning processes during robotic sensors activities.

The importance and relevance of engineering education, such as ER, is double. On the one hand, to address future needs in society, work and industry urged by the coming of technological advances and revolutions that press to develop, starting from schools, the necessary skills in future engineers to be able to anticipate sustainability problems [83] as well as to find solutions to emerging technologies in time [16]. On the other, to build digital citizens able to understand and live in a technological world surrounded by robots, the internet of things, artificial intelligence, and other engineer solutions that drive digital society.

Hence, it is time to focus on ER’s teaching-learning processes, methods, and approaches used in the pre-university education context, detect which are useful to teach and learn STEM concepts and skills and if the convergence of educational data analysis [84] as support approaches, such as Learning Analytics [85,86], can help to improve learning ER [87,88,89].

Due to the importance of the implementation and deployment of the ER curriculum, we present a systematic review of this issue below, focusing on robotics sensors’ teaching and learning methodologies in primary and secondary education. This is a systematic review of the literature published in three major databases to explore the different teaching and learning methodologies, and analytics practices applied in primary and secondary education to teach and learn the use of ER, in particular, robotics sensors. In the literature review experiences, initiatives, new curricula, the proposal of new robots, and other reviews that foster the teaching and learning of robotics sensors in primary and secondary contexts are analyzed from a learning methodologies perspective.

The STEAM learning model (where “A” represents the arts as an integral component of student learning [35,90,91,92,93,94]) incorporates technical skills [95,96], ICT competencies [97], and thought processes related to art and design into the curriculum as students learn science, technology, engineering, and math. The performing arts, including public speaking or exhibition, are useful in the communication stage of an engineering design process. Similarly, creative thinking, which is cultivated in the arts, is an integral part of the process of searching for solutions regardless of the educational area. Creativity can be learned and used in problem-solving [98,99], providing benefits in the integral learning of the student and the consolidation of concepts. Despite the availability of the STEAM learning model to enhance STEM and Arts skills in this manuscript, we position the research in STEM education results. Although we focus on STEM education research primarily, some papers using the STEAM learning model have been included in the search results.

Although there are experiences, practices, and examples of how teaching and learning methodologies around robotics sensors are applied in primary and secondary education, an inclusive and global systematic review is still missing. Therefore, this is the main contribution of this research.

## 2. Materials and Methods

We have used the Preferred Reporting Items for Systematic Reviews and Meta-Analyses (PRISMA) to conduct the systematic review [100,101]. The PRISMA approach is an update of the QUORUM [102] approach to continue improving the reporting of meta-analysis, but without limits in terms of types of studies and scientific fields. We present the results of the qualitative systematic review from manuscripts regarding aspects derived from the two research questions. We quantified, classified and analysed the found concepts in order to interpret what has been researched and published until this moment.

### 2.1. Research Questions

We define the research questions and facilitate the literature search using a specialized framework called Participants, Interventions, Comparators and Outcomes (PICO) [100,103]. This framework is commonly used in systematic reviews to formulate research questions, specially indicated in the PRISMA-P [104]. We have defined two research questions:

R1: In teaching robotics to primary and secondary school students, which pedagogical-methodological interventions result in better understanding and knowledge in the use of sensors in educational robotics? The aim is to see which teaching-learning actions are being carried out in primary and secondary education that facilitate the acquisition of concepts and skills to use robotic sensors, regarding the know-how needed to use robotics sensors correctly.

R2: In teaching robotics to primary and secondary school students, which analytical methods related to Learning Analytics processes are proposed to analyze and reflect on students’ behavior in their learning of concepts and skills of sensors in educational robotics? The aim is to see which Learning Analytics methods and techniques are being explicitly carried out.

Regarding both research questions, what we sought are individual actions of a school or collaborations between different agents focused on the teaching and learning of robotics sensors, or experiences addressed to teachers or students through the application of any teaching-learning or analytics strategies.

### 2.2. Search Strategy

In this systematic review, we have considered articles from scientific journals and papers published in conference proceedings from 2015 to September 2020. We consider this time period as adequate to carry out the review due to the recent evolution of educational robots and their sensors. Influenced by Papert’s Logo language [104,105], the LEGO Education division from its inception in 1980 to the present [106,107,108,109] evolved its products from the initial versions without motors or sensors to its latest SPIKE Prime product, with color and strength detection sensors. Concurrently, other commercial and open-source solutions appear that denote a state of maturity of the educational robotics market [110,111]. Moreover, in 2015 the maker movement was consolidated in education [112,113]. Therefore, we consider 2015 as an appropriate year to begin the collection of scientific literature due to the maturity in the market regarding robotic kits in education and the consolidation of the maker movement.

These papers deal with the execution of teaching and learning methodologies in primary and secondary education, or students, to learn concepts and skills of robotics sensors. The search has been carried out in the Web of Science and SCOPUS index databases. The keywords used were “robotics”, “sensors”, “primary”, “secondary”, “education” “analytics” and “learning analytics”, together with synonyms or derivatives of these keywords such as “school”. On the one hand, adding the word “sensors” avoided extensive results with very disparate articles and far from what we wanted to review. On the other hand, conducting such a specific search allowed us to find those articles that focused on highlighting something specific and precise about the sensors. Thanks to this narrower focus, we found some authors criticizing the sensors included in educational robotics commercial kits as being limited in either functionalities, editability, or educational aims. For instance, a search with “LEGO robotics” and “LEGO robotics light sensor” should show both papers, although “LEGO robotics” does not mean that the articles found will directly refer to sensors, which is the focus of the present research. Moreover, we excluded the search for specific trademarks, such as LEGO or Arduino, due to too many different educational robotics kits and trademarks currently available. Hence, instead of focusing on technology, we set the focus on teaching-learning strategies.

These criteria have been searched in the fields of Article title, Abstract and Keywords. Table 1 and Table 2 shows how these terms have been combined to perform the search, as well as the complete search strategy in both databases, as requested by PRISMA, so that the searches can be reproduced.

### 2.3. Inclusion and Exclusion Criteria

The papers sought had to review teaching and learning methodologies from the last 5 years used to learn concepts and skills related to robotics sensors in primary and secondary education. Thus, papers that did not focus on this topic were excluded. This process was developed in three stages, at the end of which 24 articles were assessed for eligibility and used in the present work. All of the four authors have participated in parallel in all of the phases, including searching, selecting and extracting data, in order to achieve the reliability and security of the process as recommended by PRISMA.

### 2.4. Trial Flow/Selection Process

**Figure 1:**
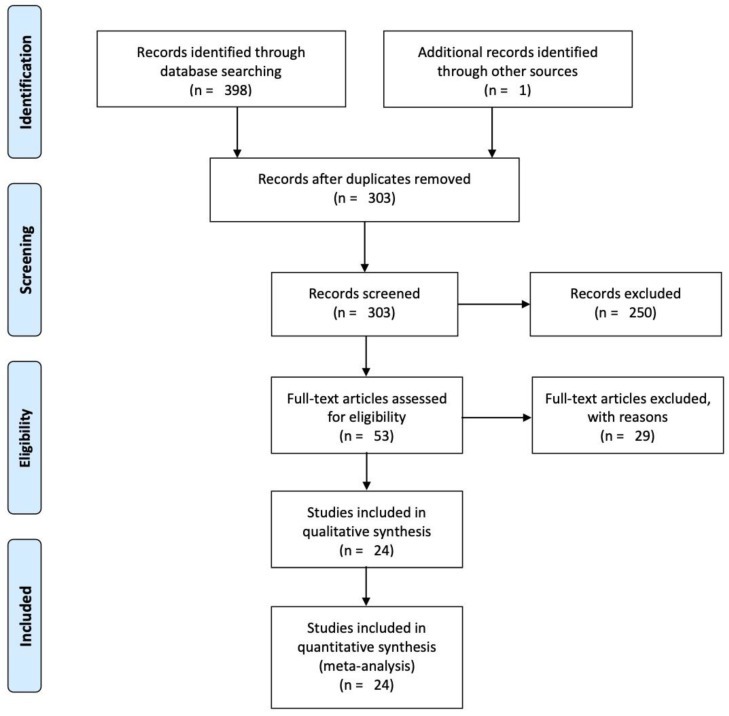
Flow diagram to show study-selection process.

A total of 784 articles were obtained in the search, 386 of which were excluded once those from 2015 and beyond were selected. From the 398 resulting articles, 97 were removed due to duplication. Next, those that did not contain the concepts searched for (robotics sensors and primary or secondary education, or robotics sensors and learning analytics) in their title, as keywords or in the summary, in any of their combinations and derivatives listed in Table 1 and Table 2 were excluded. After that, 250 additional articles were discarded. The abstracts of the remaining 53 were then analysed to see if they covered the research questions. This led to a further elimination of 29 articles, resulting in a total of 24 final articles that were analysed in depth. Thus, data has been obtained for 24 articles, which are analysed in the following sections. This data has been refined and clarified in subsequent stages. Figure 1 shows a flow chart of the whole process.

## 3. Results

We conducted an analysis of the papers resulting from the selection process, consisting of quantitative and qualitative processing in which we provide information on the number of publications per year, the countries, the publication in journals or conferences, the classification by teaching-learning methodologies, and the classification of analytical approach used in the identification of the sample indicators. The result of this initial analysis is presented in figures and tables for easy consultation and understanding, accompanying each table with a percentage analysis of the most relevant data. The four authors reviewed the results of all the papers selected. For each of the papers, we present on the one hand, two summary tables related to teaching-learning and analysis methodologies, and on the other, a summary of the essential ideas, as well as those methods applied for their execution. All this information and data lay the basis for the subsequent discussion presented in Section 4.

### 3.1. Study Descriptors

**Figure 2:**
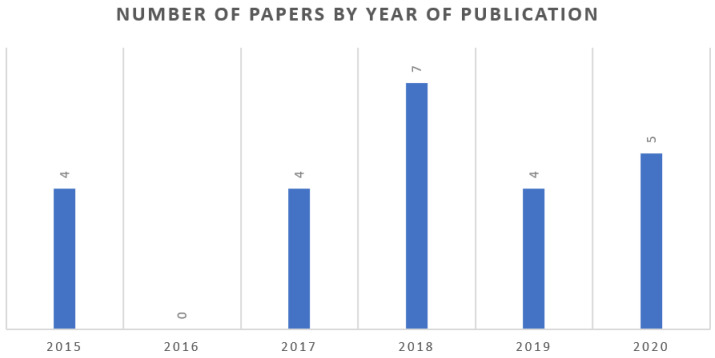
Number of published documents per year of publication (until 30 October 2020).

In regard to the number of papers by year of publication exposed in Figure 2, it can be seen an average publishing of four articles, with three significant years in the period studied: 2016 with zero papers, 2018 with seven papers, and 2020 with five papers.

Table 3a shows the summary countries of the different selected articles. It can be verified that there has been a wide participation, where four articles were published by educational institutions in the USA and four others in Spain, three are from Greece, two from Colombia and two from India, and one from each of the remaining countries in South America, Central America and Asia. Table 3b shows the medium of publication, with 10 articles sent in indexed journals and 14 in different international conferences. EDUCON is the conference with the most publications, in particular three, followed by the INTED and ISEC conferences. The journals *Computer Applications in Engineering Education* and *Advances in Intelligent Systems and Computing* presented one publication each.

We consider differences in research perspectives according to the countries of origin of the authors’ institutions. Articles from Spain, the country to which the institutions of the authors of this manuscript belong, try to solve social problems such as home-made or low-cost robots, although one of the selected articles adds more sensory capacity by using a smartphone. The USA articles deal with more recent science and technology topics such as artificial nervous systems, artificial intelligence, and algorithms applied to robotics. Articles from Greece tend to focus on the programming of robots using sensors, and those from South America tend to focus on students’ awareness of different subjects of social impact, such as electronic waste.

The first classification proposed is based on the teaching-learning strategy implemented. Reviewing the papers selected in our study, we have observed 10 different approaches that are classified in Table 4:

The most common approach used is Learning by doing with 13/24 (54.16%) experiences published, followed by Project-based learning with 9/24 (37.5%) experiences published. Seven papers have used more than one teaching-learning strategy in the learning process, and Gonzalez et al. [35] is the only study that used an approach not used in any other article (Competency-based learning).

The second classification proposed is based on the analytical approach of Educational Data Analysis (EDA). Reviewing the papers selected in our study regarding data analysis, we can observe two different approaches that are classified in Table 5:

Half of the papers identified and studied have addressed issues related with data clustering, in particular using machine learning methods such as Elbow Method based in k-means clustering. The other 50% focuses on data plotting and the calculus of perceptual analysis of collected data. 

### 3.2. Main Data and Conclusions of Each Study

In this final subsection, we present a qualitative analysis of the 24 selected papers. Table 6 and Table 7 present the papers related to each research question. In Table 6, papers are organized by robotics sensors’ teaching-learning methods extracted from the reading of each paper and the main results obtained. In Table 7, papers are organized by analytics methods extracted from the reading of each paper and the main results obtained. 

Considering that Table 6 and Table 7 summarize the results of each of the studies, we supplement the data by presenting below a synthesis of our findings.

We found articles where authors present their own robotics solutions: Balaji et al. [74] present FastBot, Bellas et al. [75] present the Robobo Project, Camargo et al. [115] present a low-cost Android and Arduino-based mobile robot, Costa, Santos and Sousa [111] created the SquirlRob, and Vega and Canas [28] present the PiBoot Tool implementation. Although all of these articles present their own solutions, the objectives tend to differ. Balaji et al. studied using robotics education as a way for school students to become aware of engineering as a career choice. The goal of Bellas et al. [18] is to inspire more practical implementations of ER. Camargo et al. provide for the connection of different sensors and actuators depending on academic requirements. Costa, Santos and Sousa support teaching robotics as a multidisciplinary scientific and engineering field. Vega and Canas provide for the addition of new types of sensors compared to those commonly included in ER commercial kits.

Some articles can be categorized in new and emerging scientific disciplines, such as neurorobotics, artificial intelligence, or remote-controlled robots. West et al. [24] present an integrated teaching program where students can learn programming and robotic design from the basics to advanced levels at little or no cost. After being introduced to programming and robotics using a pre-designed curriculum, students can remotely access and operate robots in a simulated manner. Narahara and Kobayashi [43] propose a new educational framework for teaching AI and robotics in hands-on modules for beginning learners from K-12 to adult. Harris et al. [25] investigate fundamental concepts in neuroscience while using a do-it-yourself robot built from generic parts.

The common objective of these different studies is improving the learning environments in which robotics is taught. On the one hand, allowing students to learn STEM and soft-skills concepts in different degrees of depth. On the other, to offer robotic solutions adapted to each learning need. Hartigan and Hademenos [65] merge physics and engineering concepts in a practical and hands-on engineering activity. The proposal from Jawaid et al. [60] fosters skills such as critical thinking, problem-solving, independent learning, and collaboration, and also supports learning specialized technical information. The goal of Karalekas [52] is to provide students with an open platform to learn mechatronics concepts from introductory to advanced levels. Narahara and Kobayashi [43] support the acquisition of basic programming and electronics knowledge and skills. Plaza et al. [57] conduct a workshop introducing participants to the robotics world. Rothe [63] teaches programming, algorithms, sensor technology, and robotics. Serrano and Juarez [73] develop skills for coding hardware, and artistic expression based on modeling and cardboard design.

The articles differ in the medium in which students learn robotics. Different authors carry out the study using workshops, such as Harris et al. [25], Plaza et al. [110], Stiehm et al. [118], Vega and Canas [28]. Other authors conducted their studies in courses within curricula, such as Foukarakis and Syrris [114], Jawaid et al. [60], Rothe [63], Stiehm [118], and Vega and Canas [28].

The contributions of these studies vary as well. Some articles describe the development and implementation of new robotic platforms, others can be grouped within novel scientific fields. In general, all authors seek the transmission of STEM knowledge, where some stand out for facilitating other types of skills or knowledge such as collaborative work or creativity.

## 4. Discussion

The research questions of this review of 24 papers on how robotics sensors are taught in primary and secondary education contribute to the learning of primary and secondary education students as future citizens empowered in scientific and technical skills.

Robotics, science, and technical-scientific practice, in general, have become increasingly common in both personal and industrial context [34,35]. In both social contexts, we see how artificial intelligence (AI) is integrated into different processes such as security forces and facial recognition in the field of security or detection or even prevention of disease in the field of public health. Robotics has been integrated into the workplace for many years as well, but AI has opened up new avenues and possibilities and fostered its application in many industries. The Internet of Things is also a growing trend where robotics and AI will be mixed in the near future, where drones, autonomous cars, and smart cities will be commonplace.

Faced with this present and future scenario, policymakers have understood that introducing scientific concepts into pre-university education will be key to addressing the future needs of society and industry [39], in line with the 2030 Agenda for a Sustainable -and Driven by Technology- Future [16]. These are the reasons why STEM learning plays a key role in primary and secondary education. It is therefore hoped that this education will help develop citizens committed to digital society and capable of leading future sustainable change. All the articles presented in our bibliographic review seek to engage and prepare our students in this regard.

ER [119] aims to teach science, technology, engineering, and math through activities using robots, their sensors, and their programming. Moreover, arts and humanities education are also included in these learning objectives, where STEM becomes STEAM. However, in this manuscript we will refer to STEM. To facilitate these teaching and learning goals, we analyze 24 papers to identify the most common and most effective educational practices for teaching-learning the use of robotic sensors, and how these can be aided through the analysis of educational data.

Of the 24 articles analyzed, we detected at least 10 teaching-learning methodologies: Learning by doing, Project-based learning, Challenge-based learning, Problem-solving, Discovery learning, Competency-based learning, Collaborative learning, Adventure-based learning, and Simulation-based learning. From this analysis, we extract that teaching-learning processes based on the Learning by doing methodology are the most widespread in use, as it appears in 54.16% of the articles (13 of 24). Furthermore, we observed that the second most important methodology, commonly used in dealing with unknown situations, is the Project-based learning methodology, which appeared in 37.5% of articles (9 of 24). The rest of the methodologies range from 4% to 8% occurrence in the articles. All articles consider as a learning goal the construction of a robotic device, where the sensors and the programming of the robot are fundamental for its achievement. Therefore, Piaget’s and Papert’s constructivist theories are present in all articles [35,51,52].

From the results of the articles, we conclude that building a robotic device by outlining goals to be achieved offers students the opportunity to learn to be more autonomous, to work both individually and as a team, and to acquire scientific concepts related to mathematics, mechanics, electronics, physics, technology, programming, and science in general [34,35,60,63,64,65,114]. In addition, Learning by doing or learning based on problem-solving in uncertain situations encourages students to be more aware of how problems in society can be solved with science, and to learn complex concepts requiring high cognitive ability in a relaxed way [52,57]. Moreover, neuroscience, augmented reality, virtual reality, and artificial intelligence [39,43,75,81] could play an important role in improving STEM learning [25].

With these constructivism-based teaching-learning approaches, teachers also perceive that students learn more with enjoyable activities, and are more excited, curious, and motivated, and acquire better STEM concepts than in an old-fashioned curriculum based on instructional lessons [115,117]. In summary, the processes that facilitate better learning of concepts and skills in the use of robotic sensors are those based on constructivist learning theory, which is practical and experiential, and competency-based approaches such as Learning by doing and Project-based learning [60,118].

Learning analytics, despite generating high interest currently among educators, is often used interchangeably with concepts such as artificial intelligence or machine learning [120]. We propose that this imprecise use of the above three concepts may explain the fact that we have found few papers related to sensor-centered learning analytics and educational robotics. However, in this work, we wanted to include those who have done research precisely in learning analytics and robotics sensors. We found only two. One expressly applies learning analytics using machine learning techniques, specifically the Elbow Method, performing a k-means clustering [51]. The other uses robotic sensors to collect environmental data [65], where students learn data literacy by analyzing the resulting data and making objective decisions. Both analytical actions are part of the foundation of learning analytics. With these two findings, we realize that there is still a long way to go in developing the intersection of two scientific fields such as learning analytics and robotics sensors. Therefore, we observe the need to continue learning analytics and ER as applied to robotics sensors as a line of research.

It is interesting to note that given the strong entry of commercial robotics kits, new software and hardware platforms for educational robots are appearing which are more open, more modular, and more economically accessible [73,110,111,116]. These new initiatives are moving away from a closed robot and black box [24,28] format to an open and customizable one. The new proposals have arisen for different reasons. They say that with commercial robotics kits you can learn basic notions, but that they do not allow you to bring practice closer to reality in that they fall short in showing students the real possibilities of robots. Others state reasons to justify the need for modular, open robots which use general programming languages such as Python and universal hardware or sensors, without specifying the specific brand and model of the robot. They, therefore, aim for an open ER where the software and hardware are interoperable with other robotic solutions, unlike commercially available robotics kits. Other authors point to the high prices of commercially available robotics kits, which in certain educational contexts make them impossible to acquire. All of these aspects have been observed in the current lines of research.

As a final point to conclude this review, we must highlight the fact that the 24 articles found do not present any counter argument *against* the use of educational robotics or problem-based learning. All of the experiences discussed in the literature focus on the positive. For example, the use of robots at home can foster relationships between adults and young participants. In addition, robotics education can increase attention and motivation and facilitate the learning of different concepts and skills such as mechanics, electronics, physics, technology, programming, and science in general. However, the literature related to teaching-learning methodologies presents negative results when misused or incorporated into the curriculum at inappropriate times. Therefore, it is worth exploring possible difficulties when adopting two of the most used strategies in the selected literature, Learning by doing and Project-based learning, since they are present in 90% of articles found. We caution that the idea that the use of these teaching-learning strategies in conjunction with educational robotics only leads to positive results is false. Below we present some of the counterarguments for Project-Based Learning.

As stated by David: “*The core idea of Project-based Learning is that real-world problems capture students’ interest and provoke serious thinking as the students acquire and apply new knowledge in a problem-solving context. The teacher plays the role of facilitator, working with students to frame worthwhile questions, structuring meaningful tasks, coaching both knowledge development and social skills, and carefully assessing what students have learned from the experience*” ([121], p. 80). This statement implies that teachers have the skills and knowledge necessary to implement PBL and meet learning objectives. One of the mistakes Kugle points to [122] is that PBLs can take place both inside or outside classrooms. However, when PBL extends participation time outside of class, students do not enjoy the process. Hence, poor planning of the learning experience in terms of project length, focus, or topic can produce negative outcomes [123,124].

Other possible issues with PBL regards into the language used to conduct the learning experience. Students often find that PBL, when not introduced in students’ native language, may become too challenging because of the types of activities involved. Hence, students have the feeling that they do not have enough communicative competence to complete these types of activities [125], which can also lead to lower motivation and engagement.

As we illustrated with Project-based learning, different aspects of the teaching-learning strategies must be considered and well planned, such as the needs and competencies of the teachers or students [126,127] as well as external concerns to education, such as the inability to purchase technology due to limited availability of financial resources, since these issues may lead to negative results [124].

We have to acknowledge some limitations in our review. We have used only two databases for the selection of papers and considered articles and conference papers in the very specific context of robotics sensors and teaching-learning methodologies. We are sure that a more generalist angle would have provided more results, which may be explored in future research. However, the scope of this research has allowed us to discern which are the most used teaching-learning methodologies, which pedagogical theories have been applied. We conclude that despite its steadily increasing importance, the analysis of learning in the area of robotic sensors is an almost unexplored path of research to follow. The hardware of the educational robotics kits themselves is a limiting factor. Commercial kits do not allow modifications, so extracting data from robotics sensors is not easy. Extracting data from the robotics software is likewise difficult. These limitations make conducting quantitative research challenging. Perhaps these issues may explain the lack of research using data analysis methods applied to data extracted from robotics sensors.

Finally, we point out different lines of research, which merit further exploration. On the one hand, we see the need to focus on research in Learning Analytics and ER applied to robotic sensors, due to its potential for enhancing the learning experience, and the relatively few publications we have found dealing with this phenomenon. On the other hand, we consider that it is necessary to further research the use open-source for robotics sensors and robotics in general. This research line in open-source robotics can enable new and advanced sensors, foster new learning possibilities, and reduce the price of educational robotics kits. Artificial Intelligence also requires attention to its uses in educational robotics to help introduce new STEM concepts and skills other than those commonly found in standard curricula.

## Figures and Tables

**Table 1:** Search terms and fields for Research Question 1.

Database	Search Terms
WOS complete searches	Search 1: TS = (robotics AND sensors AND secondary) OR TS = (robotics AND sensors AND primary)
	Search 2: TS = (robotics AND sensors AND secondary)
	Search 3: TS = (robotics AND sensors AND school)
	Search 4: TS = (robotics AND sensors AND “high school”)
	Search 5: TS = (robotics AND sensors AND education)
SCOPUS complete searches	Search 1: TITLE (robotics sensors) AND KEY (primary)
	Search 2: TITLE (robotics sensors) AND KEY (secondary)
	Search 3: TITLE (robotics sensors) AND KEY (school)
	Search 4: TITLE (robotics sensors) AND KEY (“high school”)
	Search 5: TITLE (robotics sensors) AND KEY (education)

**Table 2:** Search terms and fields for Research Question 2.

Database	Search Terms
WOS complete searches	Search 1: TS = (robotics AND sensors AND learning AND analytics)
	Search 2: TS = (robotics AND learning AND analytics)
	Search 3: TS = (robotics AND sensors AND analytics)
	Search 4: TS = (robotics AND analytics)
SCOPUS complete searches	Search 1: TITLE (robotics sensors) AND KEY (“learning analytics”)
	Search 2: TITLE (robotics) AND KEY (“learning analytics”)
	Search 3: TITLE (robotics sensors) AND KEY (analytics)
	Search 4: TITLE (robotics) AND KEY (analytics)

**Table 3:** Researcher countries and publication in journals/conferences.

Reference	Research Country	Reference	Journal/Conference
[25,34,43,65]	USA	[63,64,110]	*EDUCON*
[28,57,75,110]	Spain	[60,73]	*Computer Applications in Engineering Education*
[52,64,114]	Greece	[35,75]	*Advances in Intelligent Systems and Computing*
[35,115]	Colombia	[111,114]	*INTED*
[24,74]	India	[24,34]	*ISEC*
[51]	Italy	[52]	*Sensors*
[111]	Portugal	[51]	*Frontiers in Robotics and AI*
[39]	Suisse	[25]	*Frontiers in Neurorobotics*
[116]	Brazil	[65]	*The Physics Teacher*
[117]	Costa Rica	[28]	*Electronics*
[60]	Pakistan	[74]	*Procedia Computer Science*
		[117]	*Latin American Computing Conference*
		[115]	*International Conference of Education, Research and Innovation*
		[116]	*Latin American Robotics Symposium*
		[39]	*IEEE Int. Conf. on Robot & Human Interactive Communication*
		[57]	*Frontiers in Education*
		[43]	*SIGGRAPH Asia*
		[118]	*International Mechanical Engineering Congress & Exposition*
	(a)		(b)

**Table 4:** Classification of the papers selected by type of teaching-learning strategy implemented: (A) Learning by doing, (B) Project-based learning, (C) Challenge-based learning, (D) Problem solving, (E) Discovery learning, (F) Competency-based learning, (G) Collaborative learning, (H) Adventure-based learning, and (I) Simulation-based learning.

Authorship (Year) [Reference]			Type of Document						
	A	B	C	D	E	F	G	H	I
Balaji et al., (2015) [74]	X								
Bellas et al., (2018) [75]	X			X					
Camargo et al., (2015) [115]		X							
Costa, Santos, & Sousa, (2018) [111]	X								
Fonseca & Hernandez, (2018) [117]			X						
Foukarakis & Syrris, (2018) [114]		X							
Gonzalez et al., (2020) [35]						X			
Harris et al., (2020) [25]	X								
Hartigan & Hademenos, (2019) [65]		X							
Jawaid et al., (2020) [60]		X					X	X	
Johal et al., (2019) [39]	X								
Karalekas, (2020) [52]	X								
Karaman et al., (2017) [34]		X	X						
Narahara & Kobayashi, (2018) [43]	X								
Plaza et al., (2017) [110]	X								
Plaza, et al., (2019) [57]	X								
Rothe, (2015) [63]	X	X							
Scaradozzi et al., (2020) [51]		X							
Serrano & Juarez, (2019) [73]		X							
Sklirou, (2017) [64]		X		X	X				
Stiehm et al., (2015) [118]				X					X
Teixeira, Bremm, & Roque, (2018) [116]	X								
Vega, & Canas, (2018) [28]	X								
West et al., (2017) [24]	X								

**Table 5:** Classification of the papers selected by type of EDA strategy implemented: (A) k-means clustering (Elbow Method), and (B) Data Plot.

Authorship (Year) [Reference]	A	B
Hartigan and Hademenos, (2019) [65]		X
Scaradozzi et al., (2020) [51]	X	

**Table 6:** Summary of robotics sensors’ teaching-learning methodologies (based on the classification in Table 3) and main results of the papers selected.

Authorship [Reference]	Methodologies (Based on Table 3)	Main Results
Balaji et al. [74]	A	The results show high usability of the robot used. Increased motivation of the users for engineering vocations. High satisfaction of teachers with the student’s behavior.
Bellas et al. [75]	A-D	Developed approach focused on improving interaction. Adaptation of the proposal for use in smartphones. Multi-language program system environment.
Camargo et al. [115]	B	High flexibility of the platform developed. Multi sensor connection allowed. Possibility of social PBL project development. New learning processes with high-level taxonomies implemented.
Costa, Santos, & Sousa [111]	A	Application of low-cost solutions. Combined use of smartphones and virtual sensors. Possibility to use the approach presented in the resolution of mechanical, electronics, and/or informatics challenges.
Fonseca & Hernandez [117]	C	High motivation in students and teachers. Reduction of the gender gap. Improvement of the teacher’s technology capabilities.
Foukarakis & Syrris [114]	B	High level of technical and programming skills achieved by the students. Improvement of teamwork competence. High motivation.
Gonzalez et al. [35]	F	Integration of different methodologies. The student’s autonomy level was increased using robotics for problem solving.
Harris et al. [25]	A	High level of understanding in students about concepts related to neuroscience after the workshop. Low level of usability needs further improvement.
Hartigan & Hademenos [65]	B	Identification of several needs about working in a collaborative way. Low level of knowledge to develop a first robotic approach for navigation. High level of knowledge on using teamwork to address water navigation approaches.
Jawaid et al. [60]	B-G-H	Teaching and learning processes can be improved by using PBL with the integration with CL. Adding an introductory support lecture improves the final results. The course bridges the gap between technical aspects of learning and the old-fashioned curricula of schools. The approach promotes the development of problem-solving and teamwork skills.
Johal et al. [39]	A	Using AR increases the learning outcomes in a significant way.
Karalekas [52]	A	The proposal used had a positive impact on the students. The system used helped the students in understanding sensors, actuators, and controlling systems.
Karaman et al. [34]	B-C	High level of improvement in the teamwork and technical skills of the students applying software systems that allow the mini race car to operate completely autonomously.
Narahara & Kobayashi [43]	A	It is demonstrated that AI can personalize ER solutions. It is not currently possible to run AI networks in real time as a limiting factor.
Plaza et al. [110]	A	Robotics can be used to bring together adults and children. Sharing knowledge between adults and children increases their skills and knowledge about robotics. High motivation.
Plaza, et al. [57]	A	Crumble is a low-cost robot constructed by the authors that is as easy to use and can be used at home by children and adults, Helps to understand the basics of robotics sensors and increases motivation while programming.
Rothe [63]	A-B	Starter projects increase student motivation and improve scientific skills such as programming. Feedback collection should be enhanced in a systematic and rigorous manner.
Scaradozzi et al. [51]	B	Machine Learning helps to extract students’ behavior. Two behaviors detected in testing phase: those students that make small changes and others that make bigger changes.
Serrano & Juarez [73]	B	Breaking the technological and economic gap is possible with ultra-low-cost ER. Low-cost ER introduces more motivation to study programming languages and helps increase the attractiveness of different areas of engineering to students.
Sklirou [64]	B-D-E	The use of platforms based on new ICTs helps students to better understand programming techniques and methods. Interaction is fundamental to increasing motivation and understanding.
Stiehm et al. [118]	D-I	Robotics are more motivating to students than simulations. Exchange experiences, feedback and coaching by scientific professionals is relevant to deepen contents of lectures.
Teixeira, Bremm, & Roque [116]	A	Presentations and classes are not enough to raise concern about e-waste. The use of the Arduino platform elevates motivation of young people to reuse obsolete electronics.
Vega, & Canas [28]	A	Level gap detected between pre-university science curricula and university scientific and technological degrees A new educational tool was developed.
West et al. [24]	A	Use of free and open-source software to conduct high quality low-cost ER activities. High motivation.

**Table 7:** Summary of analytical methods (based on the classification in Table 4) and main results of the papers selected.

Authorship [Reference]	Methodologies (Based on Table 4)	Main Results
Hartigan and Hademenos [65]	A	Data collection and analysis from robotic sensors improves and avoids failure in ER construction. Robotics sensors data helps students to learn and apply data analysis.
Scaradozzi et al. [51]	B	Tracking systems can be integrated into robotics commercially available kits to extract student behavior and learning paths using Machine Learning techniques such as Elbow Method (k-means).

## Data Availability

Search results can be found at https://lasalleuniversities-my.sharepoint.com/:x:/g/personal/daniel_amo_salle_url_edu/EcSPFUKCsM1JkjvJJeIUZ5gBE8RW_yoWh2LRsAaPI0-Q0g?e=6I4VNL.
